# Two cases of multidrug-resistant *Neisseria gonorrhoeae* related to travel in south-eastern Asia, France, June 2019

**DOI:** 10.2807/1560-7917.ES.2019.24.36.1900528

**Published:** 2019-09-05

**Authors:** Thibaut Poncin, Manel Merimeche, Aymeric Braille, Mary Mainardis, Cécile Bebear, Hervé Jacquier, Béatrice Berçot

**Affiliations:** 1 Infectious Agents Department, Saint Louis Hospital, Assistance Publique – Hôpitaux de Paris (APHP), Paris, France; 2 French National Reference Centre for bacterial sexually transmitted infections, Associated laboratory for gonococci, Assistance Publique – Hôpitaux de Paris (APHP), Paris, France; 3 Paris University, INSERM, IAME, Paris, France; 4 University of Bordeaux, USC EA 3671, Mycoplasmal and chlamydial infections in humans, Bordeaux, France; 5 Centre Hospitalier Universitaire de Bordeaux, French National Reference Centre for bacterial sexually transmitted infections, Bordeaux, France

**Keywords:** Gonorrhoea, antimicrobial resistance, whole-genome sequencing, high dose anti-microbial treatment, travel

## Abstract

We report two cases of multidrug-resistant *Neisseria gonorrhoeae* urogenital infection with ceftriaxone resistance in a heterosexual couple in south-western France who were successfully treated with a single, high dose of intramuscular ceftriaxone (1 g). Whole genome sequencing of isolate F91 identified MLST13871, NG-MAST1086, NG-STAR233. Patient history revealed the isolate F91 was most likely acquired during a trip to Cambodia and belongs to the successful multidrug-resistant FC428 Asian clone.

The rise of ceftriaxone resistance in *Neisseria gonorrhoeae* (GC) worldwide is a major public health problem. It is of concern that multidrug-resistant (MDR) strains lead to treatment failures, even with the recommended dual antimicrobial therapy [1].

We report a travel-related MDR-GC genital infection in a heterosexual couple with high-level ceftriaxone resistance diagnosed in south-western France.

## Case description

In mid-June 2019, a heterosexual male visited his general practitioner (GP) for back pain associated with dysuria and urethritis. Two weeks earlier, he had returned from a 2-month trip to Battambang Province in Cambodia. His GP suspected urinary tract infection and prescribed a cytological and microbiological analysis of a mid-stream urine sample. This revealed the presence of leukocyturia while urine culture was negative. He did not receive antimicrobial treatment. Two weeks later, the patient came back to his GP with his wife who had symptoms of vaginitis. At this stage, he reported having sexual intercourse with a female partner during his stay in Cambodia. Screening for sexually transmitted infections (STIs) was initiated using nucleic acid amplification tests (NAATs) (Seegene kit, Eurobio, Les Ulis, France) and culture for the detection of NG at genital sites only. NAATs of a vaginal swab (female patient) and first urine void (male patient) were both positive for GC, whereas the cultures were only positive for the vaginal swab.

Antimicrobial susceptibility testing of the strain revealed a MDR-GC isolate. Upon receiving the antimicrobial susceptibility result, the two patients were treated with 1 g of intramuscular ceftriaxone. Extragenital sites were screened using NAATs on anal and pharyngeal swabs 5 days after the treatment initiation and results were negative. The patients returned for a test of cure (TOC) 2 weeks later. Both were asymptomatic and considered cured as NAATs were negative at the three sites (anal, pharyngeal and genital sites) from which TOC samples were taken.

Both patients followed the advice to abstain from sex until TOC.

## Microbiological investigation

The GC strain, referred to as F91, was recovered from the vaginal sample after a 24 h-culture on PolyViteX agar (bioMérieux, Marcy l’Etoile, France) under 5% of CO_2_ at +36 ± 1 °C and was sent to the Associated Laboratory of the French National Reference Centre for bacterial STIs in Paris for characterisation. Identification was confirmed using MALDI-TOF mass spectrometry (Vitek MS, bioMérieux). Minimum inhibitory concentrations (MICs) of seven antimicrobials were determined by ETEST (bioMérieux) and interpreted following European Committee on Antimicrobial Susceptibility Testing (EUCAST) recommendations [2]. The presence of a beta-lactamase was screened using a nitrocefin disk test (Mast Diagnostics Ltd., Bootle, United Kingdom (UK)).

The F91 isolate showed resistance to ceftriaxone (MIC 0.5 mg/L), cefixime (MIC 2 mg/L), tetracycline (MIC 4 mg/L) and ciprofloxacin (MIC > 32 mg/L). The isolate remained susceptible to spectinomycin (MIC 8 mg/L) and to azithromycin (MIC 0.5mg/L, isolates were considered resistant for MIC greater than the epidemiological cut-off value (ECOFF) at 1 mg/L [2]). It also had a low MIC for gentamicin (MIC 4 mg/L). F91 was also found to be positive for beta-lactamase activity.

## Molecular investigation

Whole genome sequencing (WGS) and bioinformatic analysis of isolate F91 was performed as previously described [3]. DNA was extracted using Wizard Genomic DNA Purification Kit (Promega, Madison, United States (US)), and DNA libraries were prepared with Nextera XT (Illumina, San Diego, US). Paired-end, 564,319 150-bp indexed reads were obtained on a MiSeq platform (Illumina). *De novo* assembly was performed using SPAdes software version 3.13.0 [4]. Quality Assessment Tool for Genome Assemblies (QUAST) software version 5.0.2 showed that the assembly yielded 204 contigs with a N50 of 28,258 nt [5]. The contigs covered 93.4% of the GC reference strain FA1090 genome. The complete genome of the F91 isolate is available in deposited at DDBJ/ENA/Genbank under accession number VNGI00000000.

WGS data were analysed *in silico* to determine the sequence types as previously described [3,6,7]. Additional resistance markers were sought using ResFinder version 3.1.1 (https://cge.cbs.dtu.dk/services/ResFinder/).

The genomes of three available French MDR-GC isolates (F89 [8], F90 [3] and F91), as well as those from recent descriptions in Europe (H18–502, H18–209 [9] and IR72 [10]) and Japan (FC428 [11]), were aligned using Parsnp software version 1.2 and corrected from recombination [12]. A maximum likelihood phylogeny was then built to compare these seven core-genomes and visualised using iTOL version 4 [13]. Relatedness between F91 and previous ceftriaxone-resistant GC isolates was observed in the phylogenetic tree (Figure), and isolate F91 appeared to be distinct from F90.

**Figure:**
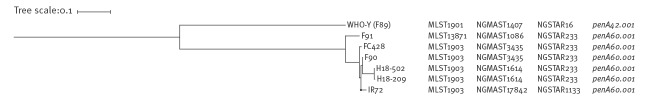
Ceftriaxone-resistant *Neisseria gonorrhoeae* isolates including the MDR-F91 isolate, France, July 2019

Core-genome maximum likelihood phylogeny based on mapping to the reference genome of the FA1090 strain and corrected for recombination. On the basis of 8,247 single nt polymorphisms, the high-level ceftriaxone resistant *Neisseria gonorrhoeae* F91 isolate is compared with previously described ceftriaxone-resistant *N. gonorrhoeae* isolates observed in Europe [3,8-10] and Japan [11]. The tree is rooted on the F89 isolate.

The isolate F91 belonged to MLST13871, NG-MAST1086 (*porB*-581, *tbpB*-21) and NG-STAR233. This isolate harbours a pJD4 plasmid carrying a *bla*_TEM-1B_ gene involved in penicillin resistance. Resistance to extended-spectrum cephalosporins (ESCs) was conferred by a mosaic *penA*-60.001 allele, encoding a mosaic penicillin-binding protein 2 previously described [14]. In addition, F91 possessed the adenine deletion in the promoter of the *mtrR* gene involved in the overexpression of the MtrCDE efflux pump, and G120K and A121D amino acid alterations in PorB1b were identified. These mutations are known to contribute to an increase in MIC values for ESCs and to MDR phenotype [15]. The two substitutions S91F and D95A in GyrA and a single S87R alteration in ParC were observed resulting in high-level ciprofloxacin resistance. No *tetM* gene was reported and tetracycline resistance was explained by a V57M substitution in the S10 ribosomal protein plus an efflux.

## Discussion

Several MDR-GC clinical isolates have been described as displaying a high-level of resistance to ESCs used for the empirical treatment of gonorrhoea. The successful FC428 clone was first observed in Japan in 2015 [11], but is now found worldwide [14], including in Europe [3,9,10,16]. This clone has also been associated with sexual contacts occurring during travels in south-eastern Asia [14] and is thought to have originated from the Western Pacific Region (WPR), with a sustained spread [9]. The woman in this case report did not travel to Cambodia and declared only having sex with her husband. She was probably infected during sexual intercourse after the return of the husband.

This is the second infection caused by a strain belonging to this clone in France in a short interval of time. At first, the F90 strain was described in Paris in November 2017, and was not associated with a history of travel [3]. However, the F91 MDR-GC isolate described here is related to a travel in Cambodia where the level of resistance of *N. gonorrhoeae* to ESCs is poorly described. Previous descriptions of the acquisition of MDR-GC in Cambodia are lacking, although it seemed conceivable according to Lahra et al. [14]. Nevertheless, ESCs resistance in the WPR is known to be high [17]. There are clear indications that travelling abroad is likely to be a risk for the acquisition of gonorrhoea [18,19], and there is growing evidence that a history of travel to certain parts of Asia may be a risk for the acquisition of MDR-GC isolates.

Some other FC428-related isolates have not been associated with a travel to certain parts of Asia. Recently, Eyre et al. described a transmission cluster in the UK related to a sexual network with links to Ibiza, Spain [9]. These observations raise the hypothesis that FC428-related isolates may have started settling in some sexual networks in Europe. Future European Gonococcal Antimicrobial Surveillance Programme (Euro-GASP) genome-based studies should provide more data to answer this question.

For the third time, a *N. gonorrhoeae* isolate displaying a high-level ceftriaxone resistance is described in France. Fortunately, the GP prescribed a 1 g dose of ceftriaxone that seemed adapted to this context of recent travel for the patients, but high dose is not yet recommended by the French STI management guidelines [20]. It was recently proposed in Japan [17] and the UK [21] to prevent the risk of treatment failures with a GC strain displaying high MICs for ceftriaxone [22].

The isolate F91, acquired during a trip to Cambodia according to patient history, belongs to the FC428 clone that originated in Asia. Although the infection was successfully treated with 1 g of ceftriaxone, the spread of this clone worldwide is of concern. A history of travel abroad should be taken systematically to inform STI management practice. In order to strengthen antimicrobial resistance surveillance, performing cultures for gonorrhoea diagnosis and antimicrobial susceptibility testing are necessary. Optimised antimicrobial therapy, early case and partner notification, treatment of partners and implementation of Point-of-care tests are needed globally when infection with an MDR-GC strain is suspected, particularly in the context of travel in south-eastern Asia. This work also highlights the need for collaborative efforts to support surveillance in countries from where MDR-GC strains have been imported.
